# Impact of COVID-19 Pandemic on Serum Vitamin D Level among Infants and Toddlers: An Interrupted Time Series Analysis and before-and-after Comparison

**DOI:** 10.3390/nu13041270

**Published:** 2021-04-13

**Authors:** Rosa S. Wong, Keith T. S. Tung, Hung-Kwan So, Wilfred H. S. Wong, Siew Yan Wong, Hing Wai Tsang, Joanna Y. L. Tung, Gilbert T. Chua, Marco H. K. Ho, Ian C. K. Wong, Patrick Ip

**Affiliations:** 1 Department of Paediatrics and Adolescent Medicine, The University of Hong Kong, Hong Kong, China; rosawg@connect.hku.hk (R.S.W.); keith-tung@connect.hku.hk (K.T.S.T.); hkso@hku.hk (H.-K.S.); whswong@hku.hk (W.H.S.W.); yanws@hku.hk (S.Y.W.); thwpaed@hku.hk (H.W.T.); tungylj@yahoo.com.hk (J.Y.L.T.); gtc510@gmail.com (G.T.C.); marcoho@hku.hk (M.H.K.H.); 2 Department of Paediatrics, Hong Kong Children’s Hospital, Hong Kong, China; 3 Centre for Safe Medication Practice and Research, Department of Pharmacology and Pharmacy, The University of Hong Kong, Hong Kong, China; wongick@hku.hk; 4 Research Department of Practice and Policy, UCL School of Pharmacy, London WC1E 6BT, UK

**Keywords:** serum 25-hydroxyvitamin D, vitamin D, infants, COVID-19, interrupted time series

## Abstract

**Background**: During the coronavirus disease 2019 (COVID-19) pandemic, the implementation of social distancing and home confinement measures may elevate the risk of vitamin D deficiency particularly for infants. This study aimed to quantify changes in vitamin D level among infants and toddlers in Hong Kong after the COVID-19 outbreak. **Methods:** We recruited 303 infants and toddlers aged 2–24 months by stratified random sampling from 1 June 2019 to November 30, 2020. Regression models were used to estimate the effect of time on infants’ serum 25-hydroxyvitamin D (25(OH)D) level overall and by age groups before and after the outbreak. Interrupted time series (ITS) analysis was performed to examine the sustained effect of COVID-19 on their serum 25(OH)D level. **Results:** The ITS results showed no immediate reduction in serum 25(OH)D level among infants, but a decreasing trend was observed in the subsequent months post-outbreak at a monthly decline rate of −6.32 nmol/L. When analyzed by age group, the magnitude of post-outbreak reduction in 25(OH)D was stronger among younger infants (aged 2–6 months). **Conclusion:** Guidelines and recommendations should be given to pregnant women and mothers to ensure sufficient vitamin D level in their infants during the COVID-19 period.

## 1. Introduction

In December 2019, an outbreak of severe acute respiratory syndrome coronavirus 2 (SARS-CoV-2) that causes coronavirus disease 2019 (COVID-19) occurred in Wuhan, China [1]. Since then, the virus has spread rapidly and put an immense burden on global healthcare systems [1,2]. While the World Health Organization (WHO) declared the COVID-19 outbreak as a pandemic on 11 March 2020 [3], people had been concerned about the impact of SARS-CoV-2 infection prior to the WHO announcement. For example, Hong Kong, a highly populated special administrative region of China, reported its first cases of locally transmitted SARS-CoV-2 infection on 23 January 2020 [4]. However, since late December 2019, the rise of stress and anxiety following media coverage of the outbreak in Wuhan [5] had begun to change the daily routine and habits of individuals and families in Hong Kong. In the past 12 months, Hong Kong has dealt with multiple waves of coronavirus infection and implemented a range of social distancing measures to suppress the transmission [6,7]. These measures include social distancing, home confinement, quarantine, isolation, and border restrictions [8]. Despite the importance of control measures during the COVID-19 pandemic, prolonged home confinement could negatively affect children’s health and development [9]. It has been posited that children undergoing long-term home confinement are more likely to be physically inactive, adopt unhealthy diets, and have limited sunlight exposure, which may put them at a greater risk of vitamin D deficiency and insufficiency [9,10,11].

Vitamin D is a steroid hormone that plays a key role in maintaining calcium homeostasis and bone health as well as boosting the immune system [12,13]. In view of its health benefits, infants and children are recommended to have at least 400IU of vitamin D intake per day [14]. Vitamin D intake is subject to the amount of environmental exposures and endogenous factors such as skin pigmentation, sun protection, ultraviolet-B (UVB) radiation exposure, and coverage of skin by clothing [13,15], and thus the level of vitamin D is known to vary widely between individuals. Previous research suggests that adequate sunlight exposure on a regular basis is sufficient for humans to meet their requirement of vitamin D [12]. People who have inadequate sunlight exposure may seek alternative sources such as oily fish, vitamin D fortified grain and dairy products, or supplements to obtain vitamin D [12,13,14,16]. However, it is particularly challenging for young infants to achieve adequate vitamin D intake because they tend to have limited sunlight exposure and dietary options [17]. Therefore, vitamin D deficiency is highly prevalent in infants and young children. A previous Hong Kong local study found that 21.9% of infants had vitamin D deficiency defined as a serum 25-hydroxyvitamin D (25(OH)D) concentration below 25 nmol/L, and the percentage can rise to 60.2% for exclusively breastfed infants [18].

The impact of COVID-19 on vitamin D intake is of particular concern to pregnant women and mothers of infants and toddlers. This is because low sun exposure and poor eating habits as a result of prolonged home confinement and social distancing can limit vitamin D intake during COVID-19, and this reduction in vitamin D intake may in turn render infants and toddlers to have an increased risk of chronic diseases including rickets, food allergies, and atopic dermatitis later in life [13,19,20,21]. To date, only one study has examined the effect of pandemic-related confinement on vitamin D level among children aged 6–84 months and its focus was on the initial outbreak of COVID-19 [11]. Furthermore, although the study addressed seasonality by comparing 25(OH)D levels in the same month before and after the pandemic, it did not take UVB radiation exposure into account, given that the ultraviolent (UV) index can vary across months and years. As the pandemic continues to evolve, there is a need for research using more robust methods such as the interrupted time series (ITS) design to investigate whether the pandemic has a sustained effect on vitamin D level in infants and toddlers. ITS is considered one of the strongest quasi-experimental designs for evaluating the effectiveness of public health interventions and health policies, as the design allows for a pre-post comparison within the same population while accounting for temporal trends and variation in other risk factors, and hence no control population is required for such evaluation [22]. In this study, we performed both simple before-and-after comparisons and ITS analysis to examine the trends of vitamin D level in infants and toddlers over the period of June 2019 to November 2020 overall and by age groups. We hypothesized that there would be delayed reductions in serum 25(OH)D concentration among infants and toddlers following the initial outbreak of COVID-19. This could be because changes in nutritional intake and lifestyle patterns in response to the containment measures might gradually come into effect and thus result in stepwise changes in vitamin D levels over the course of the pandemic.

## 2. Materials and Methods

### 2.1. Study Population

This study analyzed the data of infants and toddlers aged 2–24 months recruited by stratified random sampling in different districts of Hong Kong. Mother-infant dyads randomly selected from the attendance list were approached and recruited by trained research staff during their visit to the Maternal and Child Health Centres. Infants with any major congenital malformations, being born premature, or with low birth weight were excluded from this study. During the period of June 2019 to November 2020, a total of 303 mother-infant dyads were approached and provided informed consent to participate in this study. Upon obtaining informed consent, mothers were asked to report the sex and age (in months) of their infant. Peripheral blood samples were collected using tubes containing a clot activator from the infants by a well-trained phlebotomist. An incentive of a 200 HKD (approximately 25.6 USD) supermarket voucher was given to the participants upon the completion of the study assessment.

### 2.2. Outcomes and Covariates

The primary outcome was the vitamin D level of the infant indicated by the total serum 25(OH)D concentration. Serum was extracted from the collected peripheral blood samples to determine the vitamin D level using the liquid chromatography-tandem mass spectrometry (LC-MS/MS) method. When compared to the traditional competitive immunoassays, the LC-MS/MS method has been widely recognized for high specificity and precision [23]. Information on the participants including their age (in months) and sex was obtained at the time of blood taking. The daily means of UV indices were obtained from the Hong Kong Observatory and matched with the date of visit of the participants. The UV index is a measure that reflects the exposure level of human skin to UV radiation, on a scale of 0 (Low exposure) to 10+ (Extreme exposure) [24,25]. The Hong Kong Observatory is the government department responsible for weather monitoring and forecasting, as well as assessing radiation levels in Hong Kong.

### 2.3. Data Analysis

Descriptive statistics were summarized using frequencies and percentages for categorical data (gender) and using mean (SD) for continuous data (age and serum 25(OH)D level). The “pre-outbreak” period was defined as 1 June 2019 to 30 November 2019 and the “post-outbreak” period was defined as 1 June 2020 to 30 November 2020. We also divided our sample into two age groups: young infants (2–6 months) and older infants (7–24 months) based on the presumption that infants may have obtained vitamin D from a greater variety of food groups after six months of age. We implemented two strategies to analyze trends before and after the COVID-19 outbreak. Prior to each analysis, data were plotted graphically to detect trends to confirm the model building strategy. First, regression models were used to generate (a) within-group estimates by examining the effect of time in months on infants’ serum 25(OH)D level overall and by age groups before and after the outbreak and (b) between-group estimates by regressing serum 25(OH)D levels against the pre/post-outbreak group status, adjusting for age, sex, UV index, and month of assessment. Second, given that the change in serum 25(OH)D level, if any, could be gradual as the COVID-19 outbreak worsens, we tested whether infant serum 25(OH) D trends changed after the onset of COVID-19 compared to before COVID-19 using interrupted time series (ITS) analysis which is a quasi-experimental method to determine whether pre- and post-intervention data patterns are different from each other [26]. In this study, the ITS model estimated the baseline slope before the outbreak onset time point (pre-outbreak trend), the change in slope from the baseline trend to the post-outbreak trend (change in level immediately following the onset of the outbreak), and the immediate change associated with the outbreak time point (post-outbreak trend). Trends were approximated to be linear when discontinuities occurred. To check whether the data met the requirements of ITS design, we first plotted the data and visually compared the trend before and after the COVID-19 outbreak. We assumed the linearity of the trend lines within each segment. In addition, we used the Durbin–Watson (DW) test to check for autocorrelation, with a value close to 2 indicating no evidence for autocorrelation [27]. Furthermore, we also considered models adjusting for age, gender, and UV index that might influence serum 25(OH)D levels. All statistical tests and 95% CIs were two-sided; p values less than 0.05 were considered statistically significant. Analyses were done and figures were obtained using R version 3.6.3 [28].

### 2.4. Ethical Approval

The research protocol was approved by the Institutional Review Board of the University of Hong Kong/Hospital Authority Hong Kong West Cluster Research Ethics Committee (UW 13-055) and the Department of Health. Mothers provided written informed consent for themselves and on behalf of their infants.

## 3. Results

Our study sample included 303 infants and toddlers aged 2–24 months, of which 183 were recruited before the outbreak and 120 were recruited in the post-outbreak period. 140 were aged 2–6 months, and 163 were aged 7–24 months. Table 1 shows their demographic characteristics. Infants and toddlers recruited in the post-outbreak period generally had lower serum 25(OH)D levels than those recruited before the outbreak. The pre-post mean difference in serum 25(OH)D levels was higher in the older age group compared to the young age group. The proportion of mothers having ever breastfed in the past week prior to the survey was similar before and after the outbreak (pre-outbreak: 31.0% vs. post-outbreak: 33.3%).

Table 2 shows tests of trends for serum 25(OH)D level among infants and toddlers recruited in the pre- and post-outbreak periods. Within-group analyses showed an increasing serum 25(OH)D trend among older infants recruited in the pre-outbreak period, although it did not reach significance after adjusting for the UV index. On the other hand, among young infants (i.e., those aged 2–6 months) recruited following the outbreak, serum 25(OH)D levels were found to decline significantly at a rate of −13.39 nmol/L per recruitment month (95%CI −23.99 to −2.80). In addition, we performed between-group analyses and found that compared to those recruited in the pre-outbreak period, infants and toddlers recruited in the post-outbreak period showed significantly lower serum 25(OH)D levels (full adjustment model −10.79; 95%CI −16.89 to −4.69; *p* < 0.001), particularly among those at older ages (full adjustment model −13.34; 95%CI −20.45 to −6.22; *p* < 0.001).

**Figure 1:**
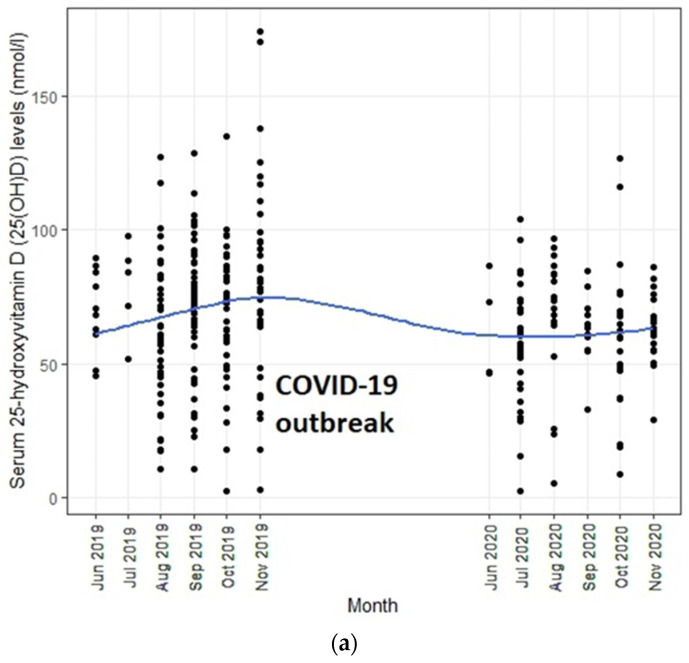

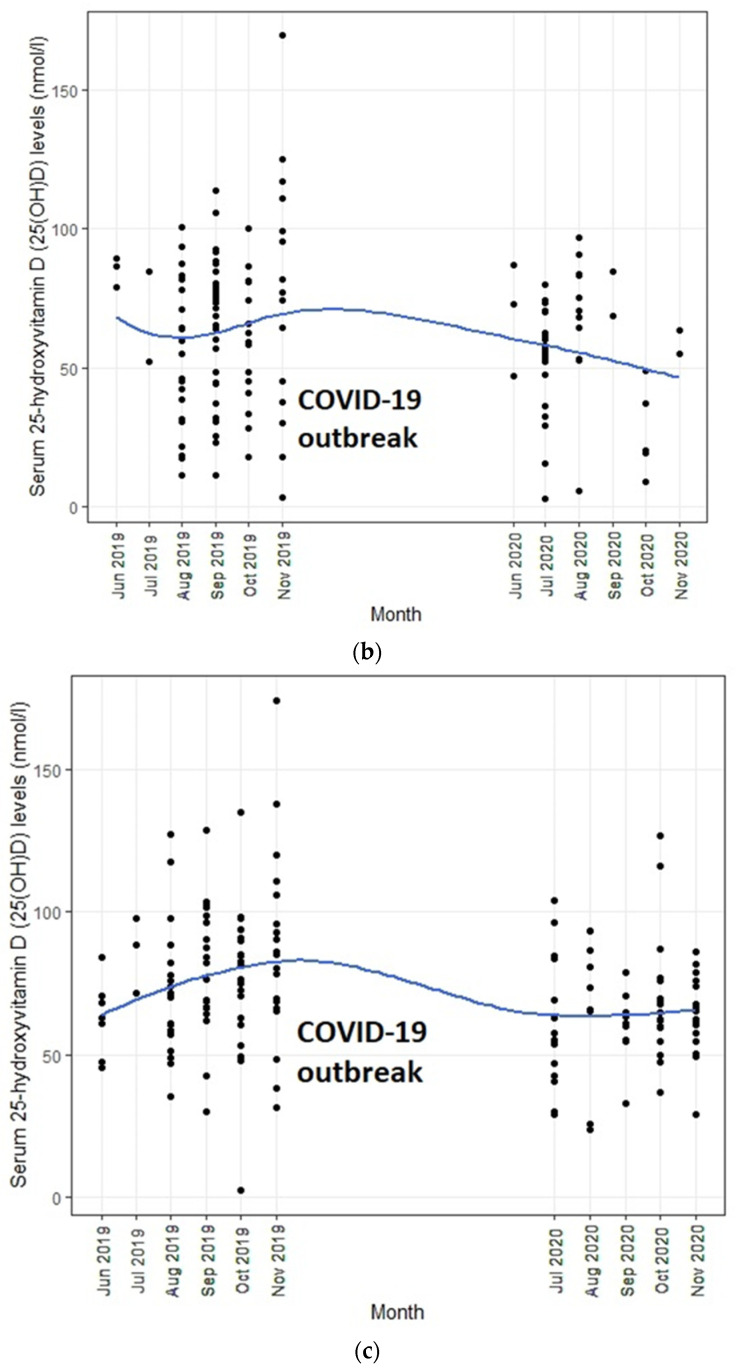
Serum 25(OH)D levels in infants (**a**) overall; (**b**) aged 2–6 months; (**c**) aged 7–24 months recruited between 1 June 2019 and 30 November 2020.

Table 3 shows the full results of the final ITS models for the overall sample and by age groups. Overall, we found no autocorrelation in the model (Durbin–Watson statistic 1.90, autocorrelation coefficient 0.047, *p* = 0.306). The pre-outbreak trend of serum 25(OH)D level in infants was positive but did not reach significance after adjusting for UV index. There were no immediate changes in serum 25(OH)D levels in infants and toddlers following the COVID-19 outbreak. However, serum 25(OH)D levels continued to decline in the subsequent months, and this decreasing trend reached significance after adjusting for age, sex, and UV index (post-outbreak monthly trend −6.32 nmol/L; 95%CI −11.95 to −0.69). Similar findings were observed for young infants. Although serum 25(OH)D levels also showed a decreasing trend following the outbreak in older infants, it did not reach significance. Furthermore, the magnitude of these post-outbreak serum 25(OH)D declines was much higher in young than older infants (young infants: post-outbreak monthly trend −9.02 nmol/L vs. older infants: post-outbreak monthly trend −1.91 nmol/L). These trends are illustrated in Figure 1a–c.

## 4. Discussion

The objective of this study was to estimate the impact of the COVID-19 outbreak on vitamin D levels among infants and toddlers recruited over the period of June 2019 to November 2020. Although the COVID-19 outbreak caused no immediate reduction in serum 25(OH)D levels of infants and toddlers in Hong Kong, we found evidence of a progressive decline in their serum 25(OH)D levels over the course of social distancing and home confinement during the COVID-19 pandemic. This study extends previous work examining the impact of pandemic-related confinement on vitamin status among young children [11]. In the face of a major and large disease outbreak, insufficient intake of nutrients particularly for young infants can occur. Older infants, compared to young infants, may have more options to obtain vitamin D and thus benefit more from stable serum 25(OH)D levels during the same period perhaps by adopting a high vitamin D diet that offsets the loss due to reduced sunlight exposure during the pandemic. However, as solid food is usually introduced at about six months old [29], decreases in vitamin D level over the course of the pandemic are plausible among infants younger than six months old.

In this study, within-group regression analysis showed an increasing trend of serum 25(OH)D levels, particularly for those aged 7–24 months recruited in the pre-outbreak period (June to December 2019), but these monthly increases became subtle after adjusting for UV index, perhaps because vitamin D levels in older infants are more affected by levels of exposure to UVB [30]. Previous research has demonstrated that those infants with limited exposure to UVB radiation tend to have lower 25(OH)D levels due to inadequate endogenous production of vitamin D in the skin [31]. Other possible factors that influence vitamin D synthesis include, but are not limited to, frequency of outdoor activities, coverage of skin by clothing, sun protection, skin pigmentation, being born prematurely, and parental knowledge regarding vitamin D [13,18,32,33,34]. Furthermore, for infants who are transitioning or have transitioned to solid food, their vitamin D level could be influenced by dietary patterns [11]. However, being born prematurely would not affect the results of this study because this was listed as one of our subject exclusion criteria.

Another finding of note pertains to the differences in vitamin D intake options between older and young infants. The overall between-group regression analysis showed that infants and toddlers in the post-outbreak group on average had a significantly lower serum 25(OH)D level than those in the pre-outbreak group, particularly for those aged 7–24 months. This could be because older infants may have other ways such as outdoor activities and sun exposure to obtain vitamin D during the pre-outbreak period. However, as a result of the COVID-19 outbreak, a series of home confinement and social distancing policies had been implemented in Hong Kong since January 2020 to contain new waves of virus transmission [35]. Indoor confinement places restrictions on outdoor play time and potentially reduces the intake of vitamin D for these older infants. On the other hand, as young infants generally have limited physical and motor capacities to play outdoors and often have skin covered up by clothing, their vitamin D levels mainly depend on feeding practice regardless of whether a pandemic has occurred, and thus the post-outbreak change in sun exposure may have limited direct effects on young infants’ vitamin D levels. To our knowledge, only one study on COVID-19-pandemic-related confinement conducted in the Guangzhou area of southern China has examined changes in 25(OH)D levels in children aged 6–84 months. The study found that reduced sunlight exposure during confinement was associated with lower 25(OH)D levels among children aged 3–6 years, and the 25(OH)D levels appeared to decline with increasing age [11]. In line with this finding, the present study supports the same notion that pandemic-related confinement has negative effects on children’s vitamin D levels and extends existing evidence by assessing the pre- and post-outbreak serum 25(OH)D levels among infants younger than six months old.

Another strength of this study is the use of the interrupted time series analysis method to test the immediate and gradual effects of the COVID-19 outbreak on vitamin D levels among infants and toddlers. We observed no significant immediate change in the serum 25(OH)D levels among infants and toddlers in the early stage of the COVID-19 outbreak. However, the serum 25(OH)D levels decreased steadily among the infants and toddlers as the coronavirus continued to spread. This resulted in a monthly decrease of about 9 nmol/L in the serum 25(OH)D levels among young infants during the post-outbreak period. Furthermore, the rate of decline for infants at six months old or below was found to be nine times faster than that for older ones. The findings suggest that pandemic-related confinement can have delayed effects on individuals’ vitamin D levels particularly for young infants. As the World Health Organization and the Centers for Disease Control and Prevention recommend infants to be exclusively breastfed for at least six months since birth [36], exclusive breastfeeding is commonly adopted for infants at six months old or below. Previous research indicates that infants receiving only breast milk are more likely to have vitamin D insufficiency and deficiency [37]. Our findings further indicate the potential of a higher prevalence of vitamin D insufficiency and deficiency among exclusively breastfed infants during the COVID-19 pandemic. This is because exclusively breastfed infants only have few options to obtain vitamin D including previous placental transfer, human milk, and sunlight exposure [38]. Prolonged home confinement and limited outdoor activities during the pandemic might cause low levels of vitamin D in pregnant women and new mothers. Previous studies have found evidence of a high correlation between maternal and cord blood 25(OD)D levels [39]. Hence, it is possible that the vitamin D stores of infants who are born during the COVID-19 period may start at a lower level than those who were born in the pre-outbreak period. It has been posited that the relatively low amount of vitamin D in human milk is insufficient to protect exclusively breastfed infants against vitamin D deficiency especially when their sunlight exposure has had been limited [40]. However, research shows that high-dose vitamin D supplementation can enhance the 25(OH)D levels of healthy lactating mothers to a level that provides adequate vitamin D intake to breastfeeding infants even when both the mother and infant have had limited sunlight exposure [41]. It is therefore important to provide regular vitamin D assessment and supplementation to both mothers and infants in times of disease outbreak such as COVID-19 wherein lifestyle and dietary changes can possibly alter nutritional status. There should also be increased efforts to educate pregnant women and mothers about the importance of vitamin D sufficiency, natural sources and supplementation of vitamin D, and alternative ways to obtain adequate vitamin D while practicing social distancing and being confined at home during the pandemic.

This study has several limitations. First, the present study did not assess the serum 25(OH)D levels in the same infants at multiple time points. In addition, the sample size of our study was considered small particularly for the ITS analysis, and data time points were restricted between June and November; hence, we adopted two analysis methods to estimate the COVID-19 impact. Although the time series and forecasting approaches used in this study permit us to make credible inferences about the association between the COVID-19 outbreak and serum 25(OH)D levels among infants and toddlers in Hong Kong, the quasi-experimental design limits our ability to draw any causal conclusions about this association. The data would have been more informative if we could track the infants’ vitamin D levels over the pandemic period. Second, the interrupted time-series design allows us to explore changes in vitamin D status after the outbreak but cannot provide information about the mechanisms that contribute to a reduction in serum 25(OH)D levels during the post-outbreak period. Finally, the serum vitamin D levels in infants could change significantly with age because of the variations in their eating and feeding practices. Notably, the range of age 7 to 24 months in this study might be too wide in comparison with the younger group. Studies with bigger samples should specify narrower ranges for age group such as 7–12, 13–18, and 19–24 months. Future research might also consider including objective assessments of dietary and physical activity patterns and other events or factors that occurred during the COVID-19 period which might help elucidate changes in vitamin D levels among infants and toddlers during this period.

## 5. Conclusions

Vitamin D is particularly important for growth, bone mineralization, and immunomodulation during infancy and early childhood. However, infection control measures such as social distancing and home confinement during the COVID-10 pandemic appear to have negative influences on the intake of vitamin D among infants and children. Our findings provide support for the delayed effects of pandemic-related policies on vitamin D levels particularly among young infants. Given the profound impact of COVID-19-induced lockdown on lifestyle habits and nutritional status, special attention should be given to pregnant women and infants during this unprecedented period, and routine provision of vitamin D assessment and supplementation to these vulnerable groups is also recommended.

## Figures and Tables

**Table 1:** Subject characteristics.

	Overall (n = 303)		2–6 Months (n = 140)		7–24 Months (n = 163)	
Age (months), mean (SD)	10.42 (6.37)		4.54 (1.52)		15.47 (4.24)	
Gender, n(%)						
Male	147 (48.51)		70 (50.00)		77 (47.24)	
Female	156 (51.49)		70 (50.00)		86 (52.76)	
Serum 25(OH)D level (nmol/L)	n	mean(SD)	n	mean(SD)	n	mean(SD)
*Before the COVID-19 outbreak*	*183*	*70.58 (28.53)*	*93*	*64.17 (29.58)*	*90*	*77.20 (25.94)*
June 2019	10	69.51 (15.46)	3	84.93 (5.46)	7	62.90 (13.37)
July 2019	5	78.86 (17.71)	2	68.15 (22.98)	3	86.00 (13.12)
August 2019	44	61.02 (26.59)	23	51.84 (27.13)	21	71.08 (22.52)
September 2019	53	71.70 (24.68)	34	66.97 (24.45)	19	80.16 (23.35)
October 2019	36	68.60 (25.82)	15	58.85 (23.32)	21	75.57 (25.76)
November 2019	35	82.04 (38.43)	16	76.54 (43.54)	19	86.67 (34.06)
*After the COVID-19 outbreak*	*120*	*61.00 (20.85)*	*47*	*55.60 (22.55)*	*73*	*64.48 (19.04)*
June 2020	4	63.35 (19.82)	4	63.35 (19.81)	0	0.00 (0.00)
July 2020	38	56.85 (21.08)	22	52.51 (19.16)	16	62.83 (22.73)
August 2020	19	66.28 (24.88)	11	67.65 (24.96)	8	64.39 (26.34)
September 2020	13	63.33 (12.47)	2	76.59 (11.47)	11	60.92 (11.48)
October 2020	25	60.49 (26.27)	5	26.81 (15.87)	20	68.91 (21.10)
November 2020	21	62.46 (12.72)	3	57.83 (4.94)	18	63.23 (13.53)

**Table 2:** Effect of time on serum 25(OH)D level in infants and toddlers in Hong Kong before and after the COVID-19 outbreak.

	Before the COVID-19 Outbreak (June to November 2019)		After the COVID-19 Outbreak (June to November 2020)			
	Within-Group		Within-Group		Between-Group ^a^	
	β (95%CI)	*p*-Value	β (95%CI)	*p*-Value	β (95%CI)	*p*-Value
Overall (n = 303)						
Unadjusted	3.36 (0.28, 6.44)	0.033	0.78 (−1.60, 3.16)	0.519	−9.57 (−15.53, −3.62)	0.002
Adjusted for age and sex	3.30 (0.28, 6.32)	0.032	−0.63 (−3.25, 1.98)	0.631	−11.16 (−17.07, −5.26)	<0.001
Additional adjustment for UV	3.03 (−3.51, 9.58)	0.361	−5.91 (−13.78, 1.97)	0.140	−10.55 (−16.64, −4.45)	<0.001
Additional adjustment for UV and month	-	-	-	-	−10.79 (−16.89, −4.69)	<0.001
2–6 months (n = 140)						
Unadjusted	2.93 (−2.11, 7.96)	0.251	−2.58 (−7.49, 2.33)	0.296	−8.57 (−18.28, 1.14)	0.083
Adjusted for age and sex	2.85 (−2.17, 7.86)	0.263	−3.25 (−8.21, 1.70)	0.193	−9.35 (−19.23, 0.52)	0.063
Additional adjustment for UV	1.88 (−8.53, 12.29)	0.720	−13.39 (−23.99, −2.80)	0.015	−7.24 (−18.79, 4.31)	0.217
Additional adjustment for UV and month	-	-	-	-	−7.21 (−18.81, 4.39)	0.221
7–24 months (n = 163)						
Unadjusted	3.66 (−0.003, 7.33)	0.050	0.61 (−2.41, 3.62)	0.690	−12.72 (−19.91, −5.53)	<0.001
Adjusted for age and sex	3.88 (0.22, 7.53)	0.038	−0.40 (−3.55, 2.74)	0.798	−12.83 (−20.00, −5.65)	<0.001
Additional adjustment for UV	4.13 (−4.36, 12.61)	0.336	3.55 (−8.71, 15.80)	0.565	−12.75 (−19.94, −5.56)	<0.001
Additional adjustment for UV and month	-	-	-	-	−13.34 (−20.45, −6.22)	<0.001

^a^ June to November 2019 (reference group) all coefficients are unstandardized. A *p*-value < 0.05 denotes statistical significance.

**Table 3:** ITS analysis of trends in serum 25(OH)D level in infants and toddlers in Hong Kong before and after the COVID-19 outbreak.

	Model 1		Model 2 ^a^		Model 3 ^b^	
	β (95%CI)	*p*-Value	β (95%CI)	*p*-Value	β (95%CI)	*p*-Value
Overall (n = 303)						
Time	3.36 (0.58, 6.15)	0.018	3.31 (0.59, 6.03)	0.017	1.67 (−2.45, 5.80)	0.425
COVID-19	−26.00 (−53.01, 1.01)	0.059	−12.67 (−39.93, 14.59)	0.361	26.08 (−52.26, 104.42)	0.513
Time since COVID-19	−2.59 (−6.60, 1.43)	0.206	−4.24 (−8.26, −0.22)	0.039	−6.32 (−11.95, −0.69)	0.028
2–6 months (n = 140)						
Time	2.93 (−1.73, 7.58)	0.216	2.96 (−1.67, 7.61)	0.208	−0.67 (−7.31, 5.96)	0.841
COVID-19	3.13 (−45.60, 51.86)	0.899	−0.24 (−48.86, 48.38)	0.992	78.04 (−35.20, 191.27)	0.175
Time since COVID-19	−5.50 (−12.99, 1.99)	0.149	−5.25 (−12.76, 2.26)	0.169	−9.02 (−17.98, −0.06)	0.048
7–24 months (n = 163)						
Time	3.66 (0.39, 6.93)	0.028	3.85 (0.63, 7.08)	0.020	5.16 (−0.18, 10.49)	0.058
COVID-19	−28.84 (−63.80, 6.12)	0.105	−23.87 (−59.06, 11.32)	0.182	−57.56 (−172.98, 57.87)	0.326
Time since COVID-19	−3.06 (−7.89, 1.78)	0.214	−3.85 (−8.71, 1.00)	0.119	−1.91 (−9.91, 6.09)	0.638

^a^ Adjusted for age and sex; ^b^ further adjusted for UV; all coefficients are unstandardized. A *p*-value < 0.05 denotes statistical significance.

## Data Availability

The data that support the findings of this study are available from the corresponding author upon reasonable request.

## Abbreviations

UV	Ultraviolet
25(OH)D	25-hydroxyvitamin D
COVID-19	coronavirus disease 2019
WHO	World Health Organization
SARS-CoV-2	severe acute respiratory syndrome coronavirus 2
ITS	Interrupted time series
